# Removal of *Penicillin G* from aqueous solutions by a cationic surfactant modified montmorillonite

**DOI:** 10.1016/j.mex.2019.08.019

**Published:** 2019-09-10

**Authors:** Heshmatollah Nourmoradi, Ali Daneshfar, Sajad Mazloomi, Javad Bagheri, Safora Barati

**Affiliations:** a Biotechnology and Medicinal Plants Research Center, Ilam University of Medical Sciences, Ilam, Iran; b Department of Environmental Health Engineering, School of Health, Ilam University of Medical Sciences, Ilam, Iran; c Department of Chemistry, Faculty of Science, Ilam University, Ilam, Iran; d School of Medicine, Gonabad University of Medical Sciences, Gonabad, Iran; e Department of Analytical Chemistry, Islamic Azad University, Ilam, Iran

**Keywords:** Application of removal of *Penicillin G* from Aqueous Solutions by a Cationic Surfactant Modified Montmorillonite, Adsorption, Montmorillonite, *Penicillin G*, Aqueous solution

## Abstract

Nowadays, antibiotics have been found in the effluents of many pharmaceutical industries and hospitals, sanitary sewage, surface water and groundwater. The purpose of this study was to investigate the possibility of using Hexadecyl Trimethyl Ammonium Bromide modified montmorillonite (HDTMA-Mt) as an inexpensive and suitable adsorbent for the removal of *Penicillin G* from aqueous solutions. The experiments were conducted in a batch system. The effects of different variables including surfactant loading onto the clay, solution pH, contact time, adsorbate concentration and temperature were investigated on the removal of *Penicillin G*. Surface properties of the clay were evaluated using X-ray diffraction (XRD) and Fourier-transform infrared (FTIR) techniques. Various isotherms (Langmuir and Freundlich) and kinetics (pseudo-first order, pseudo-second order and intraparticle diffusion models) of adsorption were studied for the data evaluation. The findings indicated that the sorption capacity of the modified clay was found to be 88.5 mg/g over 60 min contact time at pH 9. The pseudo-second kinetic (R^2^ = 0.999) and Freundlich isotherm (R^2^ = 0.915) models best fitted the experimental data of *Penicillin G* by the adsorbent. The negative values of ΔG at higher temperature and positive value of ΔH showed the endothermic and spontaneously sorption of the drug by the clay. It can be concluded that the modified clay can be considered as a cheap and eco-friendly sorbent for the removal of *Penicillin G* from water and wastewater.

## Graphical abstract

**Figure:**
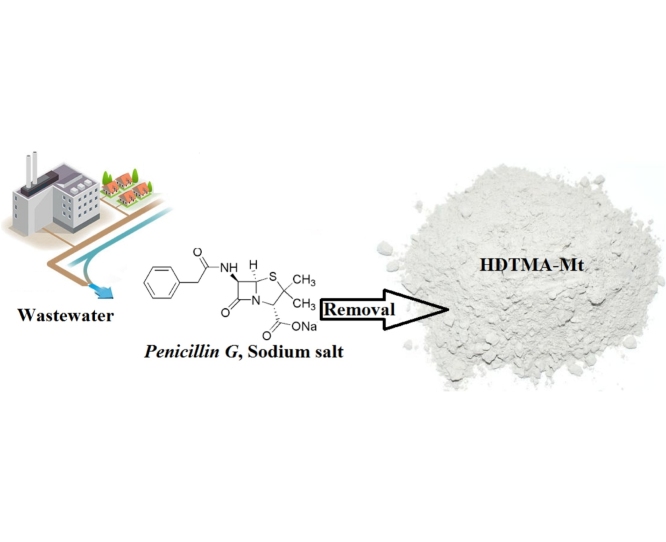

**Specifications Table**

**Table:** 

Subject area:	Environmental sciences
More specific subject area:	Wastewater treatment
Protocol name:	Application of removal of *Penicillin G* from Aqueous Solutions by a Cationic Surfactant Modified Montmorillonite.
Reagents/tools:	The effects of different parameters such as surfactant loading rate onto the clay, solution pH, contact time, adsorbate concentration and temperature were explored on the sorption of *Penicillin G* from aqueous phase
Experimental design:	The sorption process was conducted in batch mode by 100 mL of the drug solutions containing the clay adsorbent
Trial registration:	No applicable
Ethics:	No applicable

**Value of the Protocol**

**Table:** 

• HDTMA-Mt as a cost-effective and eco-friendly adsorbent was used for the removal of *Penicillin G* from aqueous media. • Various kinetics, isotherms and also thermodynamic data obtained by this study are useful for designing and planning sorption system of *Penicillin G* removal by the modified clay in water and wastewater. • The pseudo-second kinetic (R^2^ = 0.999) and Freundlich isotherm (R^2^ = 0.915) models best fitted the experimental data of *Penicillin G* by the adsorbent.

## Description of protocol

### Data

**Fig. 1:**
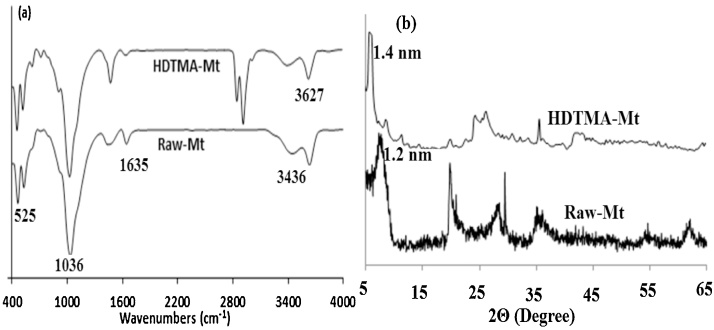
FTIR (a) and XRD (b) patterns of raw montmorillonite (raw-Mt) and hexadecyl trimethyl ammonium bromide (HDTMA) modified montmorillonite (HDTMA-Mt).

**Fig. 2:**
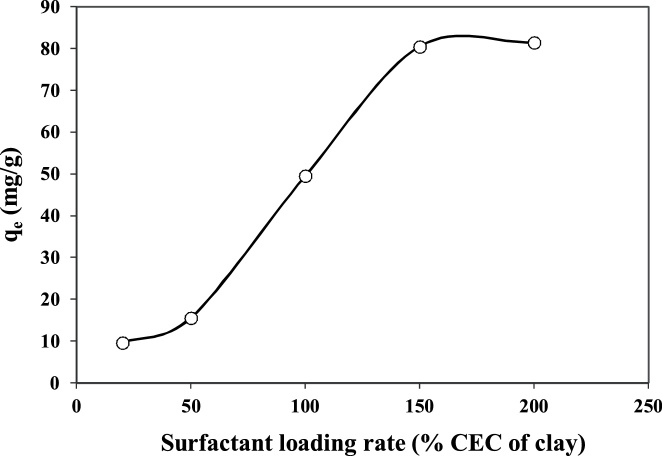
The effect of surfactant loading rate on the removal of *Penicillin G* by clay (adsorbent dose = 0.1 g, C_0_ = 120 mg/L, contact time = 240 min, pH = 7.0).

**Fig. 3:**
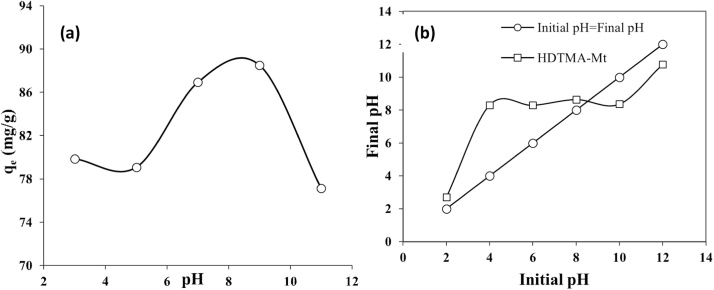
(a) The effect of solution pH on the removal of penicillin G by clay (adsorbent dose = 0.1 g, surfactant loading = 150%, C_0_ = 120 mg/L, contact time = 240 min), and (b) pH_zpc_ of the adsorbent.

**Fig. 4:**
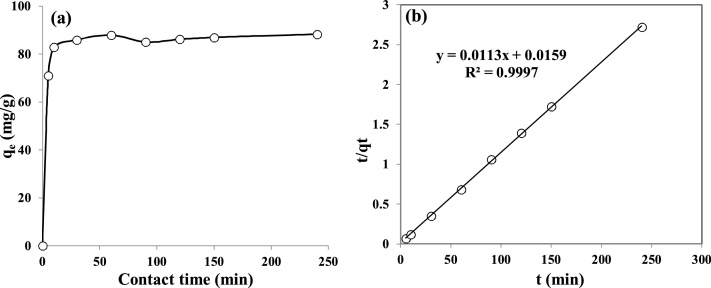
(a) The effect of contact time on the removal of *Penicillin G* by clay and (b) Pseudo second order kinetic model (adsorbent dose = 0.1 g, surfactant loading = 150%, C_0_ = 120 mg/L, pH = 9.0).

**Fig. 5:**
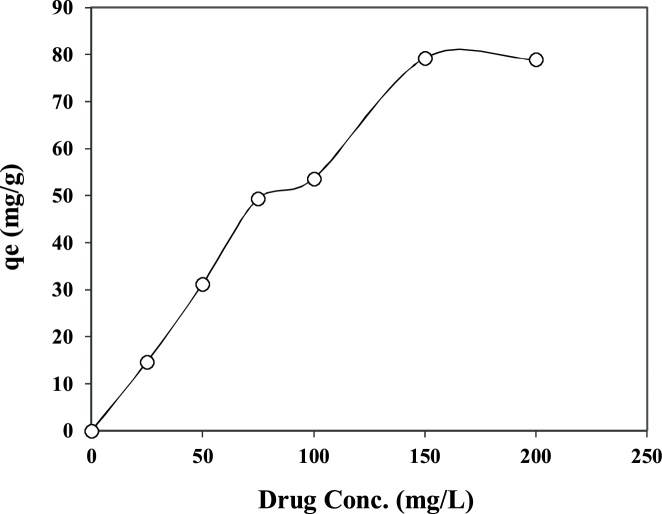
(a) The effect of drug concentration on the removal of *Penicillin G* by clay (adsorbent dose = 0.1 g, surfactant loading = 150%, contact time = 60 min, pH = 9.0).

**Fig. 6:**
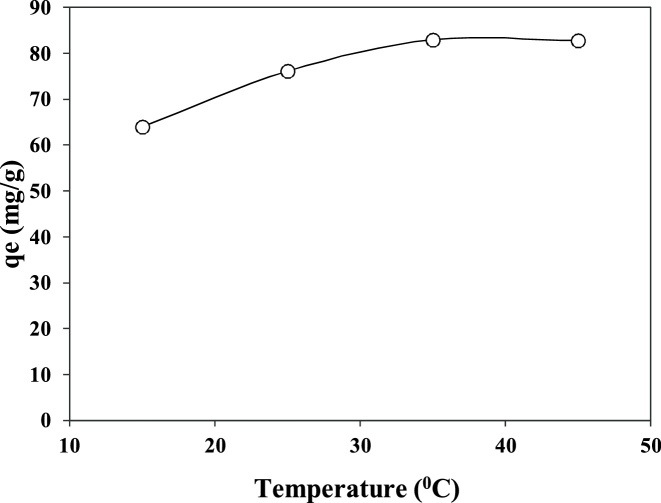
The effect of temperature on the removal of *Penicillin G* by clay (adsorbent dose = 0.1 g, surfactant loading = 150%, contact time = 60 min, drug Conc. = 150 mg/L, pH = 9.0).

Concern about water pollution by different pollutants from human activities is becoming greatly increased in many developing and developed counties [[1], [2], [3], [4]]. *Penicillin G* or *Benzylpenicillin* is one of the common antibiotics, which is used successfully for prevention and treatment of many bacterial infections in humans and animals [5,6]. After use by human and animals, up to ninety percent of the antibiotics may be excreted via urine and faeces into sewage and eventually in the water bodies and environment [7,8]. Antibiotics in environment may also increase the problem of development and spread of antibiotic resistance, posing a potential threat to public health, since they can be released into the environment after their use [9,10]. Nowadays, antibiotics have been found in the effluents of many pharmaceutical industries and hospitals, sanitary sewage, surface water and groundwater [11]. Therefore, development of an effective method is greatly desired for the treatment *Penicillin G* from effluents in order to protect the public health in communities. Adsorption can be considered as an effective method for the uptake of many contaminants from domestic and industrial effluents [7]. Table 1 lists the main physical and chemical characteristics of *Penicillin G*. The experimental runs were carried out according to Table 2. The FTIR and XRD patterns of the raw and modified montmorillonite are shown in Fig. 1. The influences of different parameters including loading rate of surfactant onto the clay, solution pH, contact time, drug concentration, and temperature are presented in Fig. 2, Fig. 3, Fig. 4, Fig. 5, Fig. 6. For Fig. 3(b), at pH value lower than pHzpc, the adsorbent surface becomes positively charged, while at pH more than pHzpc, the adsorbent surface is negatively charged. The pHzpc value obtained for the adsorbent was 8.5, that is the pH at which the curve crosses the line pH_initial_ = pH_final_. Also, Table 3 shows kinetics, isotherms and thermodynamic studies applied in this research. The values of various kinetics and isotherms models and thermodynamic are listed in Table 4, Table 5, Table 6, respectively.

**Table 1:** Physical and chemical properties of *Penicillin G*.

Parameter	Value
Chemical formula	C_16_H_17_N_2_O_4_S.Na
Molecular structure	
Molar mass (g/mol)	356.4
Appearance	powder, white
Density (g/cm^3^)	1.41
Solubility in water (mg/mL)	100
Maximum absorption wavelength (nm)	313

**Table 2:** The experimental runs for the removal of Penicillin G by montmorillonite.

No	Exprimental run	Surfactant loading rate (% CEC clay)	Solution pH	Contact time (min)	Drug Conc. (mg/L)	Temperature (°C)
1	Effect of surfactant loading onto clay	20–200	7	240	120	25
2	Effect of solution pH	a*	3–11	240	120	25
3	Effect of contact time	a*	b*	0–240	120	25
4	Effect of drug Conc.	a*	b*	c*	25–200	25
5	Effect of temperature	a*	b*	c*	d*	15–45

a*, b*, c* and d* are the optimum values obtained at previous stage for the above-mentioned factors.

**Table 3:** The kinetics, isotherms and thermodynamic models used in this study.

Model	Equation	Plotting	Obtained parameters	
			Slope	Intercept
Isotherms				
Langmuir [18]	$\frac{C_{e}}{q_{e}}=\frac{C_{e}}{Q_{m}}+\frac{1}{bQ_{m}}$	$\frac{C_{e}}{q_{e}}\;vs.\;{\;C}_{e}$	$\frac{1}{Q_{m}}$	$\frac{1}{bQ_{m}}$
Freundlich [19,20]	$lnq_{e}=ln{\;k}_{f}+\frac{1}{n}\text{ln}{\text{C}}_{\text{e}}$	$lnq_{e}\;vs.\;\text{ln}{\text{C}}_{\text{e}}$	$\frac{1}{n}$	$ln{\;k}_{f}$
Kinetics				
Pseudo-first order [21,22]	$\ln\left(q_{e}-q_{t}\right)=\ln q_{e}-k_{1}t$	$\ln\left(q_{e}-q_{t}\right)\;vs.\;t$	$k_{1}$	$\ln q_{e}$
Pseudo-second order [23,24]	$\frac{t}{q_{t}}=\frac{1}{k_{2}q_{e}^{2}}+\frac{t}{q_{e}}$	$\frac{t}{q_{t}}\;vs.\;t$	$\frac{1}{q_{e}}$	$\frac{1}{k_{2}q_{e}^{2}}$
Intraparticle diffusion [8]	${\;q}_{t\;}=K_{id}t^{\frac{1}{2}}+C$	${\;q}_{t\;}\;vs.\;t^{\frac{1}{2}}$	$K_{id}$	C
Thermodynamics [25]	ΔG=-RT ln k, $\text{ln}k=\frac{\Delta S}{R}-\frac{\Delta H}{RT}$	$\text{ln}k\;vs.\;\frac{1}{T}$	$\frac{\Delta H}{R}$	$\frac{\Delta S}{R}$

C_e_ = equilibrium concentration (mg/L), q_e_ = sorption capacity (mg/g), Q_m_ = maximum sorption capacity (mg/g), b = Langmuir constant, k_f_ and n = Freundlich constant, q_t_ = sorption capacity at time t, t = contact time (min), k_1_, k_2_ and k_id_ = kinetic constants, ΔG = Gibbs free energy (kJ/mol), R = gas constant, T = temperature (K), k = thermodynamic constant, ΔS = entropy (kJ/mol K), ΔH = enthalpy (kJ/mol).

**Table 4:** The values of various kinetics parameters in this study.

Pseudo-first order			Pseudo-second order			Intraparticle diffusion		
q_e_	K_1_	R^2^	q_e_	K_2_	R^2^	K_id_	C	R^2^
6.02	0.012	0.489	90.90	0.008	0.999	0.86	77.09	0.491

**Table 5:** The values of isotherms parameters in this study.

Langmuir			Freundlich		
Q_m_ (mg/g)	b (L/mg)	R^2^	K_f_	n	R^2^
200.0	0.009	0.577	2.6	1.22	0.915

**Table 6:** Thermodynamic parameters for the removal of *Penicillin G* by HDTMA-Mt.

q_e_ (mg/g)				ΔG (kJ/mol)				ΔH	ΔS
288 K	298 K	308 K	318 K	288 K	298 K	308 K	318 K	(kJ/mol)	(J/mol K)
63.98	76.13	83.0	82.8	0.54	0.11	−0.33	−0.76	13.09	43.56

## Experimental design, materials and method

### Materials

Montmorillonite clay was purchased from Laviosa Co (Italy). *Penicillin G* sodium salt (<96%) antibiotic and HDTMA surfactant (≥99%) were provided from Sigma Aldrich Co (USA). Other chemicals including HCl and NaOH were obtained from the Merck Co (Germany). To adjust solution pH, HCl and NaOH (0.1 M) were used in the experiments. The stock solution (1 g/L) of *Penicillin G* was weekly made by distilled water and stored at refrigerator (4 °C). The desired working solutions were prepared using dilution of the solution.

### Modification of montmorillonite

First, 30 g of montmorillonite clay was dissolved in one liter of distilled water. The suspension was mixed at room temperature (25 °C) using a mechanical stirrer (600 rpm for 24 h), and after that it was centrifuged (6000 rpm for 15 min). The above process resulted in the separation of impurities such as quartz and iron oxide in the bottom of clay. After centrifugation, the impurities were separated and the treated clay was dried (60 °C for 24 h). The purified clay was finally ground and sieved to 125 μm [12]. The above purification action increased the cation exchange capacity (CEC) from 86 meq to 108 meq per 100 g of the clay. The obtained clay was finally modified by different loading rates of HDTMA and used in the experiments.

### Study design

The experiments in this study were conducted in batch mode at room temperature. All the testes were carried out by 100 mL of the solution containing *Penicillin G* into 250 mL Erlyn myer flasks via 0.1 g of the modified clay. The suspensions were mixed by an orbital shaker (250 rpm). The concentration of the drug was measured into the clear supernatant after centrifugation (6000 rpm for 15 min) by an UV/Vis spectrophotometer at maximum absorption wavelength of 313 nm. All the experiments were respectively performed according to Table 2. Eq. (1) was used to calculate the adsorption capacity [13,14]:

(1) $${\text{q}}_{\text{e}}=\frac{\left({\text{C}}_{0}-{\text{C}}_{\text{e}}\right)\text{V}}{\text{m}}\;\;$$

where q_e_ (mg/g) is adsorption capacity of clay, C_0_ and Ce (mg/L) are the initial and residual concentrations of the contaminant in the solution, respectively. V (L) is the volume of the solution and m (g) is the clay mass used [[15], [16], [17]].

### Analytical methods

The FTIR spectra of the raw-Mt and HDTMA-Mt were specified by a FTIR spectrophotometer (JASCO, FT/IR-6300, Japan) in wavelengths of 400–4000 cm^−1^. The crystallite structure of the adsorbents was also determined via a X-ray diffractometer (Bruker, D8ADVANCE, Germany) by Ni filtered Cu Kα radiation (1.5406A^o^). The concentration of *Penicillin G* into the cleared solutions was determined by an UV/Vis spectrophotometer (DR5000, Hach, USA).
